# The Asylums of Holland: Their past and Present Condition

**Published:** 1854-07-01

**Authors:** Daniel H. Tuke

**Affiliations:** Licentiate of the Royal College of Physicians, and Assistant Medical Officer to the York Retreat


### THE ASYLUMS OF HOLLAND: THEIR PAST AND PRESENT CONDITION.

BY DANIEL H. TUKE, M.D.,

*Licentiate of the Royal College of Physicians, and Assistant Medical Officer to the York Retreat.*

Having in the autumn of last year visited the principal asylums in Holland, and collected some information respecting their past, as well as their present condition, I conclude that a short report of them will not be uninteresting to the readers of your Journal.*

And first I will speak of the former treatment of the insane in Holland, and of the various Acts of the Legislature passed from time to time in regard to them.

The condition of the insane in Holland, half a century ago, was no less deplorable than in other countries; and I have no evidence of any movement in their favour at the period when Pinel did so much for them in France. "In our country," says Professor Schroeder van der Kolk, of Utrecht, "where no knowledge was neglected, and in which so many institutions and other proofs of our humanity and benevolence are to be found on the most magnificent scale, this most important care (that of the insane) and necessary part of humanity, has been altogether neglected, and in fact has hardly an existence. For although Boerhaave, that luminary not only of our own country, but of Europe also, had given most useful precepts in the treatment of those diseases, indicating a great and humane man—even he could not free himself from Galen's notions about black bile.

"Gaubius, however, in his first oration, 'Of the Regimen of the Mind, which is the Office of Physicians,' proved with so much eloquence, and so powerfully illustrated, the force which the body has over the mind, and the efficacy of medicine in correcting mental diseases, that I am surprised that our countrymen should have so much neglected his advice, and have been so ignorant of what other countries were effecting. What indeed formerly was the condition of maniacs in our country, is evident from those words of Swietenius, in which he relates, that there was a certain man among the Batavi celebrated in the treatment of the insane, who inflicted on these miserable creatures, when they were violent, stripes as he would have done on wild beasts, immersed them in water, chained them, and hungered them."

Political troubles interfered with the care and attention of Government, until Holland recovered her independence in 1813. In the following year—a period in England remarkable for an extraordinary reaction in favour of the insane—an Act of King William I. (Feb. 12) facilitated the admission of recent cases of insanity into the asylums, previously the receptacles for chronic cases only.

In 1816, the Government made a census of the insane, by which there appeared to be 1259 in the eight Northern Provinces—a return which had the effect of showing the necessity for a thorough inspection of all places in which lunatics were confined; no step, however, in that direction was at that time taken.

In 1825, another census was made, which gave a return of 1828 lunatics (868 men, 960 women), the population being 2,253,794, or 1 insane in 1232, Of these 1828, 702 were confined in 47 distinct places—prisons, workhouses, poorhouses, hospitals, &c.; only 23 of these abodes being houses specially for the insane; and not one really and truly adapted to their treatment.

* The writer has already comprised an account of the general result of a visit to the Continental asylums, in an essay now being published by the "Society for the Improvement of the Condition of the Insane;" but the limits of that essay did not allow of a particular notice of individual asylums.

The war with Belgium, from 1830 to 1833, appears to have again interrupted the endeavours and investigations of Government relative to lunatics. Private efforts, however, were not wanting at this period; the directors of the Utrecht asylum commenced the work of practical reformation in that establishment—and this attempt may be regarded as the *point de départ* from which all subsequent improvements in the Dutch asylums proceeded—exhibiting as it did the successful results of an improved system of treatment, and attracting the attention of not only medical men, but of the Government also, to the advantages of such a system, and the necessity of a thorough reform in the asylums in Holland. This asylum was visited in 1836 by Frederick Prince of Orange, who expressed his astonishment at the order and tranquillity prevailing there.

To Professor van der Kolk, the physician to the asylum, belongs the merit of having pioneered the way in this noble cause; and not content with his practical labours at the asylum, he made an inspection of the condition of the Dutch asylums, and in 1837 delivered an oration, "*De debita cura infaustam maniacorum sortem emendandi eosque sanandi, in nostra patria nimis neglecta.*" In this he eloquently depicts the deplorable condition of the insane in his country even at that time, and earnestly adjures his countrymen to redeem their character, by making a suitable provision for their insane population. Indeed Professor van der Kolk must be regarded as the Pinel of Holland. The following quotations from his address, cannot fail to be of interest to the reader; they exhibit the condition of the Dutch asylums in 1837, and bear ample witness to the zeal and humanity of the orator:—

"This [deplorable] state of things was common to us and other countries, but that which gives us so much cause for regret is, that at the time when, in most parts of Europe, the medical treatment of the insane was greatly ameliorated, in *our* country there was scarcely any improvement. Now, there do exist here and there private asylums in which the insane are kept and humanely treated, but they are generally committed to the care of private individuals, entirely ignorant of the medical art, of which notwithstanding these diseases form the most delicate and difficult part. But in other places (and these by far the greatest number) they are shut up in narrow cells and dungeons, no better than in the time of Swietenius; or they lead a miserable life, mixed up with prisoners and thieves. Should ever any return cured from these receptacles (which is hardly ever the case), the fact affords a most forcible testimony—the strongest proof indeed—what great obstacles, greater in short than the disease itself, Nature is sometimes able to surmount. On the doors of these dungeons there might be appropriately inscribed those words which Dante inscribes on the gates of the Infernal City:—

"'Lasciate ogni speranza voi ch'entrate.'"

In English:—

"All hope abandon, ye who enter here!"

In tracing the progress of the subject, the orator alludes to an essay written by Dr. Guislain, for a prize offered by the College of Medicine at Amsterdam, and which was published by it in 1826. This first attempt to rouse public attention to the subject was frustrated, it is thought, by the magnitude and expense of the establishment proposed—which alarmed the Dutch mind. "I therefore rejoice exceedingly," he continued, "that I should have lived to see even in our country an establishment for the cure of the insane, which may be compared to those which exist in other countries. In our city, first, this was established, or rather a most pestilent cancer was changed into an institution of this salutary character. * * * We have, therefore, something to rejoice at, in which all unite who have learned the principles of charity to mankind, which are inculcated by our holy religion. But we have this additional cause of rejoicing, that, with the Divine blessing, not less than 60 persons have departed cured from our establishment during the last three years; once more in the enjoyment of the gift of reason, restored to their country, their friends, and themselves. In this also we may rejoice, for I speak to you my friends, who are united with me in the care of this establishment; we have desired no other reward for our labours but this rejoicing, this Divine blessing, and the consciousness of right actions. Let us then press forward with fresh ardour, my most esteemed coadjutors, and God will thus answer your desires. He who, inspired by love to his fellow-men, spends his time and his strength in alleviating their calamities—he has not lived in vain, he has remained at his post, and fulfilled his calling; he has obeyed the precepts of the God of mercy." * * * * *

He then refers to the large number of other institutions for the good of humanity, and alludes especially to one—

"In our time a society has been formed by men acting under the most humane feelings, and under the auspices of the King, for the benefit of thieves and other criminals; which proposes, by inducing them to amend their lives, to fit them to return to society—who does not admire such an effort? But do we see any society formed for the care of the insane, with equally disinterested motives? Yet they are contaminated with no crime. Is their cure more desperate than that of thieves? Why then do they mourn without help, without alleviation, without being thought worthy of any amendment of their lot? Why is cruelty and indignity often their portion, and this among people the most refined in manners, the most cultivated in intellect—a nation professing Christianity? What heart is there so hard that is not moved with pity at the sight and contemplation of these unhappy beings, who ought to be the objects of assistance and kindness, and not of punishment, and with the highest indignation against those who perpetrate these things—who permit them—and who regard all this inhumanity as a matter of amusement or derision?"

After stating that seclusion is not the only thing necessary, but that to this, medical and moral treatment must be added, calculated to deliver patients from this malady and restore them to their friends, he exclaims:—"Humanity—if there be any—demands it; the love of our race—if it has an existence—demands it; the Christian religion no less demands it, which represents love and charity as the highest virtues, and inhumanity and cruelty as the worst of crimes. Which of us, I ask, would not call him a wretch, and avoid him as he would any other plague, who should be so lost to a sense of humanity that he should refuse to care for a father, a brother, a son, or any other relative, or unfortunate friend depressed by heavy grief and sorrow, and deprived of all health, and not only should deny him every comfort and assistance, but also should shut him up in prison lest he should be a burden to him! Nevertheless there are and were melancholics, and these not a few, to whom this misfortune has happened. For this disease depresses the mind and the reason itself, and induces despair; which deplorable condition increases by the unkindness of men, and what is more, is rendered perpetual, until by the compassion of God and by death, their miseries and the cruelty of men are destroyed. * * * * * I have already touched on the opprobrious treatment resorted to by the rustics to whom they have been confided, of the beatings they have endured, of the chains by which they have been galled, and of the dungeons and prisons (*caveas et carceres*) in which they have been immured. With my own eyes have I seen maniacs shut up for fifty years, in caves into which neither the light of the sun nor the breath of heaven could enter. I have seen a female maniac whose joints had been rendered incapable of motion by anchylosis, consequent on being constantly retained in one position by fetters. Her mind was restored, but she deeply bemoaned the total loss of bodily power she had sustained. Who that sees such things as these can refrain from tears?"

In 1838 another census was made, resulting in a return of 1925 lunatics

(931 men, 994 women), the population being 2,583,271, or a proportion of 1 to 1336. Of these 1925, 826 were confined in 26 houses of a very wretched description; and the attention of Government was afresh directed to the necessity for stringent legislation—chiefly at the instance of the Utrecht professor. And in 1841 (May 29) an Act was passed for the purpose of reforming the abuses of asylums, which has been of so much service in achieving its object, that in ten years all unfit houses of this description have been abolished. In the same year also two Commissioners in Lunacy were appointed, one a medical man, (Professor van der Kolk), and the other a lawyer connected with the Home Department (M. C. T. Feith); it is much to be regretted, however, that they were invested with very limited powers, and were very insufficiently remunerated. Still they had full power to inspect all asylums, and report on their condition to the Minister of Home Affairs. They themselves deserve the thanks of their country all the more for having worked as they have done, notwithstanding the smallness of their salary, which rendered it necessary for them to continue their professional avocations, the one at Utrecht, the other at the Hague.

In the year following their appointment, the Commissioners made a tour of inspection, and found 923 insane persons, confined in 32 houses; those of the provinces of Limburg being now included in the number. Of these 32, only 3 (the asylums of Utrecht, Zutphen, and Deventer) deserved approval—the remainder being found totally unfit for the reception of the insane. The result of this Report was, that Government ordered a large number of them to be immediately closed, while the rest were left to the more gradual influence of the reforming Act of 29 May, 1841. Of the provisions and working of this Act, we may here briefly speak:—Art. 2 divides all residences for the insane into—A. Medical asylums for the cure of the insane, and B. Residences for the confinement of chronic cases. Now, on the report of the Commissioners of the unfitness of any house for the medical and moral treatment of the insane, they were classed under division B.; and new admissions into them were rendered extremely difficult and tedious by the regulations of the Act. On the contrary, every facility was given to the admission of cases into the medical asylums (class A).

In addition to this, Art. 3 of the Act forbade the establishment of private asylums; even two insane persons were not permitted to be received into any private house.

Such was the influence of the articles of this Act, that, on the 31st of December 1850, only six houses for the confinement of chronic cases remained, the total number in which only amounted to 51; and these the Commissioners found to be treated with kindness and attention. It appears that before long these six houses will also be closed, and that what are called "medical asylums" will then only exist.

This Act also provides by its 8th Article for the care and treatment of the insane paupers by each province; and it enacts that "the States of those Provinces in which it is found impossible or unnecessary to establish a 'medical asylum' for the treatment of the insane, shall contract for their insane with the committees of one or more medical asylums in other provinces." This article has, however, been erroneously interpreted by many of the provinces, and they have made such contracts with distant asylums, when it was their duty to have established asylums in their own provinces. Owing, therefore, to the great distance of the asylums contracted with, the number of the insane sent has been quite disproportionate to the insane population, and, as the Table will show, has left a large proportion still only provided for by their friends at home.

The following tabular account* of the number of insane in Holland, and of

* See the last Report of the Dutch Commissioners in Lunacy, 1852.

the asylums existing for their care, will be of interest to the reader. From it the proportion of the insane to the population appears to be nearly 1 in 1000, but it is more than probable that subsequent statistical inquiries will prove the insane population to be greater than this. Professor van der Kolk informed me that he believed 1 in 800 would not be too high a proportion. The table also shows that there is, properly speaking, only one provincial asylum, that of Meerenberg, for North Holland, the erection of which owes most to the philanthropic exertions and rare intellect of the Governor van Ewyck van der Bilt, and the liberal voluntary contributions of the citizens of Amsterdam.

TABLE,

*Showing the Provision of the Provincial Governments in Holland for their Insane Population, and the general Condition of the Medical Asylums on December 31st, 1850.*

| Provinces. | Population on Jan. 1, 1850. | Total of Insane, calculated at 1·1000 Inhabitants. | Insane in Asylums. | Insane *not* in Asylums. | Number of Asylums. | Situation of Asylums. | Where there is a Resident Medical Superintendent. | Where the Asylum is Provincial. |
|---|---|---|---|---|---|---|---|---|
| Drenthe . . . | 83,269 | 83 | 9 | 74 or 89 per cent. | | | | |
| Groningen . . | 188,806 | 188 | 37 | 151 „ 80 „ | | | | |
| Friesland . . . | 246,967 | 246 | 50 | 196 „ 79 „ | | | | |
| Zeeland . . . | 160,073 | 160 | 42 | 118 „ 73·8 „ | | | | |
| Limburg . . . | 205,431 | 205 | 55 | 150 „ 73·2 „ | 1 | In Maastricht. | | |
| Noord Brabant . | 396,845 | 396 | 116 | 280 „ 70 „ | 1 | „ 's Hertogenbosch. | | |
| Overyssel . . . | 216,274 | 216 | 67 | 149 „ 69 „ | 1 | „ Deventer. | 1 | |
| Gelderland . . | 370,560 | 370 | 142 | 228 „ 61 „ | 1 | „ Zutphen. | 1 | |
| Utrecht . . . | 149,347 | 149 | 81 | 68 „ 45 „ | 1 | „ Utrecht. | | |
| Zuid Holland . | 562,354 | 562 | 338 | 224 „ 38 „ | 4 | { „ the Hague. „ Delft. „ Rotterdam. „ Dordrecht. } | | |
| Noord Holland . | 476,665 | 476 | 326 | 150 „ 31 „ | 2 | { „ Amsterdam (Jews). Country seat, Meerenberg. } | 1 | 1 |
| Totals . . | 3,056,591 | 3056 | 1263 | 1793 „ 59 „ | 11 | Only one in the country. | Only in three. | Only in Noord Holland. |

Since December 31st, 1850, Friesland has opened an Asylum within the town of Francher, and to this Asylum, as also to those in Utrecht and Dordrecht, a Resident Medical Superintendent has been appointed.

Before Meerenberg was built Dr. Everts, its present superintendent, had done much at the Deventer Asylum in the care and treatment of the insane; and I must not omit to mention as deserving of all praise the efforts made under very disadvantageous circumstances by G. E. V. Schneevoogt, physician to the General Hospital at Amsterdam (Buitengasthuis), a few wards of which were devoted to the treatment of about 200 insane. Dr. Ramaer also has done much for the cause of the insane in Holland by his labours at the Zutphen Asylum, and by his published works. Credit also is due to him for having started a Psychological Journal, the first which has appeared in his country.

It was, as I have said, in the autumn of 1853 that I visited Holland. I will now—after this rapid sketch—proceed to briefly describe the asylums I saw, more particularly that of Meerenberg, which may be regarded as a model asylum in its construction as well as in its internal arrangements. The reader must excuse the imperfection of the following notes, being extracts from letters hastily written to my father, Samuel Tuke, of York.

ROTTERDAM.—"I had hoped to find at Rotterdam a recently erected asylum for the insane, and being in the first instance conducted by the commissionaire to the General Hospital by mistake, I concluded that my anticipations were correct. But my disappointment was great when I found that the splendid new building I had arrived at was not for the insane—a disappointment still further increased when I subsequently was shown the veritable asylum, an old building in the heart of the town, affording a very indifferent accommodation for its inmates.

"I was conducted over the house by the director, who, unhappily, is not a medical man; unhappily for the interests of the patients, as also for the purposes of my visit. Subsequently, however, I met Dr. Charenté, the visiting physician to the asylum, a man of kindly disposition, and very willing to give me any information in his power to communicate. It appears that a new asylum is contemplated, but the funds are not provided, nor is the plan designed.

"There are at the present time 117 insane persons in the establishment. Of these 49 are men and 68 women. Their condition was certainly not very satisfactory; the amount of restraint by means of the camisole and the coercion chair very considerable; and several of the rooms for the violent class were damp, gloomy, and in all respects unfit for the reception of a patient. At the same time, I should be very sorry to convey too unfavourable an impression; I did not see anything that could be called inhumane treatment, and many of the wards were clean, and the patients engaged in in-door employment. Owing to its locality, farming, or in fact any out-door employment, is precluded. As to the classification of the patients, it does not appear to extend beyond the noisy, and dirty; and the tranquil. Nine men and seven women are what are termed 'dirty patients.'

"I find that during the ten years ending 1850, 397 patients were admitted into the asylum. Of these 138, or 34·9 per cent. of the admissions were discharged cured—a proportion which although low as compared with many well-conducted asylums, is not so low as to justify any conclusion reflecting very unfavourably on the treatment pursued.

"Again, during the seven years ending 1851, 150 males and 147 females were admitted; and of these 58 men and 53 women were discharged cured, making a per centage of 38·7 men and 36·1 women, or a mean of 37·3, a somewhat higher proportion. Epileptics and incurable cases are admitted.

"The mortality, however, appears to have been very high, for between 1841 and 1848, 113 patients died. Now, the average number resident was 77·5,—thus giving a mean annual mortality per cent. resident of 18.* Between the years 1844-51, however, the rate of mortality was less—being only about 14 per cent.

"This asylum is one of four which provide for the insane of South Holland, the population of which in 1850 was 562,354. Of the remaining three one is

* Typhus fever is stated to have removed some.

at the Hague, one at Delft, and the other at Dordrecht. All are small asylums; it is evident, therefore, that the number *out* of asylums must be very large indeed."*

The Hague.—"This asylum I referred to in a former letter, as one of the four asylums for South Holland. It has been built two years, and is situated completely in the town,—in which circumstance, and its having no resident medical officer, it resembles the asylum at Rotterdam. In other respects, however, it is far superior. Dr. Starck and M. Eckendam are the visiting physician and surgeon. The house contains 84 patients; 27 being men, and 57 women. There are three classes as regards payment: the 1st pay 600 florins (50*l.*) per annum; the 2nd, 400 (32*l.*); and the 3rd, 250 (20*l.*) The two last classes are paupers, and are paid for by the Government. The director only receives a very small stipend; and the salary of the attendants ranges from 8*l.* to 5*l.*; so that everything is in proportion. Provisions are also much cheaper than in England.

"The general appearance of the patients was satisfactory; the dress, of the women especially, very neat; and although no carpets were to be seen, there was both in the day and bed rooms considerable evidence of comfort and cleanliness. In the work-room were a dozen patients, who appeared to be busily engaged, and to be very contented. The bed-rooms, for the most part, contained more beds than is desirable in dormitories, some having 13, others 15. There were fewer, however, in the rooms on the male side of the house. Iron bedsteads are much used. The poor patients appeared generally to lie on straw mattrasses; the rich had, in addition, a spring mattrass; the latter is certainly a great luxury, but to a stranger its great resiliency reminds him of the voyage he may have just taken across the ocean, and renews some of the qualms he had hoped to have forgotten!

"So much for the quiet patients. For the violent were provided very fair seclusion rooms, which were heated by a warming apparatus. The patient is inspected, not by means of an opening in the door, but from above. The amount of restraint was not large. I regretted, however, to see one patient fastened into a chair by leather straps attached to the legs, arms, and wrists. Padded rooms have found their way into this asylum; the pads composed of horse-hair enclosed in canvas. On the whole, I believe the treatment pursued in this asylum is humane, and that, although there is room for improvement in several respects, there is a sincere desire to pursue the means best calculated to promote the patient's recovery. It is greatly to be regretted, therefore, that the gardens or airing-courts of the asylum are much confined, and that there is no provision for agricultural employment, and no means of affording entertainments on a large scale in the open air to the patients. It is indeed deplorable that an asylum, for which but recently many thousand pounds have been spent in additional accommodations, should be situated within a town, and be thus doomed always to remain an asylum of inferior rank.

"The Catholics preponderate in this asylum. A chapel for religious worship is being built. In the Hague itself are 26,000 Protestants, 26,000 Catholics, and about 8000 Jews.

"In regard to statistics, I find that during the seven years ending 1851 (a period extending further back than the new building) 195 patients were admitted—viz., 101 men, and 94 women; of these, 40 men and 37 women, or a total of 77, were discharged cured, being a per centage of 39·6 males, 39·4 females, and a mean of 39·5. During the same period, 49 died—17 women, and as many as 32 men. Calculated on the admissions, the total per centage would be 26·1. But reckoned on the mean number resident, the annual mortality per cent. would be as follows:—Men, 19·3; women, 7·5; mean, 14·3. This shows an extraordinary mortality among the men; the per centage of recoveries is fair."

* See Table, p. 449; the information contained in which was subsequently obtained.

Meerenberg.—"To-day has been most pleasantly spent at Meerenberg, the name of the asylum for the insane of North Holland. It is beautifully situated near Haarlem, and not far from the sea; and in so flat a country as Holland, the sand hills (dunes) in the neighbourhood strike the eye very agreeably, and are highly prized by the inmates of the asylum.

"Dr. Everts is the physician in chief. He visited the Retreat, and many other English asylums, in 1848, accompanied by Dr. D. H. van Leeuwen; the result of that visit was a determination to adopt in this asylum, which was opened on their return, the principles of non-restraint. Dr. Everts speaks English, and was extremely kind in supplying me with information about the establishment. In Dr. Everts I found a man devoted with all his heart to the comfort and cure of those placed under his charge. Dr. van Leeuwen, a man like minded with, and formerly, as I have said, the colleague of, Dr. Everts, at Meerenberg, has lately left on account of his health, and has been succeeded by Dr. Persyn on the female side, and Dr. Opdorp on the male side of the house, both resident in the establishment. This proportion of medical officers contrasts but too favourably with ours in England. The effective working of thus having one medical officer with supreme authority over two medical men, who have also their respective departments, must depend on their complete co-operation; but, on the whole, I am inclined to think the system works well.

"The asylum is built in the Italian style, and consists essentially of a large quadrangle, composed of a ground-floor and upper story. The square formed by the building is divided into two equal parts, by a one-storied erection uniting the front with the back elevation; the ventilation of this area is, however, by no means deficient, the distance of the wings being too considerable to interfere with the free access of air and light. From the centre of the front elevation rises a three-storied building, devoted to administrative purposes; and on either side are the rooms occupied by patients of the paying class. Attached to these is a separate division for epileptics of the same class; it has no second story, and contains a padded room, a dormitory, and a dining-room. The lateral wings are devoted to the inferior class of patients (in regard to payment), and contain dining-rooms, baths, &c. on the ground-floor, and dormitories above. From these are now being projected (at right angles to the sides) additional wings, one for the pauper men and the other for the pauper women. One only is completed; it has wards for the convalescent and the inoffensive patients; as also nine seclusion-rooms for the violent, one of which is padded.

"The dirty patients and pauper epileptics are placed in the back elevation, the extreme ends of which are carried out in the form of the letter T; the central portion is occupied by workshops, the laundry, &c.

"A chapel will be erected at a little distance from the asylum, but a temporary one is situated in the line of buildings already referred to as dividing the quadrangle into two equal parts; here also are placed the school-room, the apothecary's shop, the store-room, and the kitchen.*

"The effect produced on the visitor's mind on approaching the asylum is very agreeable. It gives no impression of a place of confinement. The panes of glass are of larger dimensions than any I have seen; except those in the division for the violent, &c., where they are of an ordinary English pauper asylum size. In the former, the upper sash is moveable, but its descent beyond a certain point is prevented. The size of the panes, however, is such that by breaking the glass a patient so disposed could easily escape. No inconvenience, however, I was informed, has so far resulted from this arrangement. Escapes are stated to be rare, which is the more remarkable, as there is no wall around the estate, and the divisions between the airing courts are of wood. Certainly if, by a careful selection of the cases not likely to abuse the privilege, the frontage of an asylum can be provided with windows similar to those of a

* *Vide* plan at commencement of article.

gentleman's mansion, it is only right to provide such, out of regard to the feelings both of the inmates (accustomed to them) and of their friends, whose first impressions are not to be overlooked. In regard to suicides;—there has been only one since the opening of the asylum, though there are two large ponds near the house, one of which has been almost entirely made by the patients. They serve in the summer for fishing, and in the winter for skating. I understood that once only had a patient attempted self-destruction in these ponds; but she was immediately saved from a watery grave by her attendants. Then, as indirectly affecting the number of escapes and suicides, the proportion of attendants is by no means excessive, though very fair—namely 44 to 391 patients, or 1 to nearly 9. No argument, therefore, can be justly founded on the number of attendants, to show that the slight amount of restraint employed here incurs unreasonable expense. An English attendant would think himself very poorly paid at the Dutch rate of remuneration—none having more than 100 guilden a-year (8*l.* 6*s.* 8*d.*), and board.

"The number of patients now in the house is, as I have said, 391: of these, 163 are men, and 228 are women. Private asylums are not now allowed in Holland;* and all classes, as to payment, are provided for at Meerenberg; but by far the largest proportion are paupers. There are five classes;—the first paying 1000 guilden per annum (84*l.*); the second, 750; the third, 500; the fourth, 400; and the fifth, 250. This asylum, as well as others in Holland, is visited by the Dutch Commissioners in Lunacy, Professor Kolk, of Utrecht, and M. Feith, of the Hague.

"Next, as to the number in restraint. Dr. Everts is an advocate of the non-restraint system, and is most anxious entirely to abolish all mechanical forms of coercion: at the present time, however, owing to temporary circumstances which I need not detail, restraint is not altogether discontinued. But to how very slight an extent it is employed will be seen, when I state that Dr. Everts showed me a carefully kept daily register of restraint, and that, during the present month, the number ranged from 1 to 3 per diem; and that, last month, not one man was under restraint, and the number of the women averaged 1 to 2. At the time of my making my visit, there were 2 men and 1 woman restrained,—if, indeed, the latter could be called restrained, being only prevented falling out of a chair by a strap: the former were confined by camisoles. This restraint was temporary, and on no pretext whatever is it practised without the order of the superintendent. The seclusion-room (padded, or not, as is required), the strong dress, and similar means, are here employed, as substitutes for restraint, in addition to the constant surveillance, classification, and moral treatment on the part of the officers and attendants.

"As to classification, the wards are divided, first, in reference to payment into the highest, the lowest, and an intermediate class; and these wards are respectively divided according to the mental condition of the patients (as I have mentioned, in speaking of the plan of the building),—as the tranquil, the noisy, the epileptic, and the dirty. The room in which the noisy were placed was extremely gratifying—being quiet (in asylum language), and the patients clean, and as comfortable as their circumstances would admit of. The comfort and cheerfulness of the rooms are very much increased by cages of considerable size fastened to the walls, and filled with singing birds. Mirrors in some rooms, and pictures on the walls, also added very much to their home-ish appearance.

"The bed-rooms were extremely clean—very lofty; and those for the upper class elegantly furnished, and thoroughly home-like. For the pauper class the

* It is the opinion of Dr. Everts, Dr. van Leeuwen, and Professor van der Kolk, that the plan of admitting all classes of patients (as to property) into one establishment, with proper separation of the paupers from the other classes, possesses many moral and economical advantages; that it especially benefits the poor patients, and offers no real inconvenience to the rich. In Holland they consider that the English system of exclusive pauper asylums, is objectionable in many respects.

proportion of dormitories is considerable; several rooms containing eight beds, some twelve, and one I counted with fifteen. In the new wing the average number in one room is less. For the higher class the mattresses are of hair; for the lower, of fine grass, in addition to an under one of straw. The canvas-frame, &c., is employed, as in England, for the dirty class of patients. Of this class there are at present only 9 men and 16 women. The epileptics sleep on the ground floor. They number 21 on the male side, and 25 on the women's. Very minute observations are made of the circumstances attending their fits; the temperature, the state of the barometer, the hour of the day, or night, &c. Dr. Everts finds that the fits of the women are more frequent between 9 P.M. and 9 A.M. than at other times, and most frequent at midnight. On the contrary, with the men, the attacks were of more frequent occurrence in the day.

"Dr. Everts' experience in regard to the general paralysis of the insane—its relative frequency in the two sexes—its early symptoms—its fatality, &c., quite accords with that of Calmeil and the superintendents of English asylums. As to the employment of the patients, I saw abundant evidence of the attention paid by Dr. Everts to this important subject. The work-room on the women's side was truly a delightful sight—containing a large number of clean, neatly-dressed individuals, busily engaged in needlework, the expression of their countenances testifying, in a language common to all nations, of their comfort and happiness. The laundry and drying-room also afford work to the women: others are busied in making the beds, and scouring the rooms and windows—an occupation a Dutchwoman appears thoroughly to enjoy, and while so engaged seems to have adopted for the motto on her escutcheon, "Rest is rust!" You see the servants in the streets, with a pail, and a most effective hand-pump, firing away at the windows, and then, with an enormously long broom, completing the work of purification, refusing all quarter to either the dust or the spiders that may have collected there.

"Then there are workshops for the men—in which joinering, shoemaking, tailoring, &c., are carried on, under the oversight and assistance of properly-qualified persons. Much out-door employment has arisen from the new grounds requiring laying out, &c. About 100 men and 150 women are employed at the present time in various ways. But the mind is not forgotten; and in the school-room many (including idiots) are regularly taught reading, writing, drawing, &c.

"In the evenings, parties of patients are formed in various wards, and in company with the officers and attendants amuse themselves in reading, talking, singing, music, billiards, and innocent games. Thus the day closes at Meerenberg, the patients having felt, some perhaps for the first time, that they are not outcasts from society, but are the objects of kindly feeling and regard.

"As regards medical treatment, I may just state that general bleeding is very rarely employed; when it is, the cases are usually apoplectic, and not maniacal. Topical depletion by leeches and cupping are much more frequently resorted to, Dr. Everts stating that the latter would be employed at least once daily. Tunod's *ventouse monstre* is also considered of great service in deriving the blood from the spinal cord and cerebrum, and in restoring the catamenia in hysterical mania. Emetics are occasionally found of use. There were reported to be on the day of my visit 50 women and 40 men under medical treatment—many of these, of course, quite independent of the mental disease.

"The grounds are, as I have said, very beautiful. We took a walk through some magnificent avenues of elm and beech, through one of which Dr. E. conducted me to his house, which is quite distinct from the asylum. The wife and child of a pauper patient were strolling together in one of these pleasant shady walks, and were very kindly greeted by the excellent superintendent. In such a place, —— might (if anywhere) with reason exclaim, 'There is every temptation to lose one's wits!'"

In addition to the information contained in the above Letter, I may here add the following Tables, extracted from the Reports of 1850 and 1851:—

### Table I.

*Showing the number of Patients, dirty, excited, restrained, and secluded.*

| Period. | Average No. daily dirty. | | Average No. daily excited. | | No. daily in restraint. | | No. daily in restraint chair. | | Seclusion by day. | | Seclusion by night. | |
|---|---|---|---|---|---|---|---|---|---|---|---|---|
| | M. | W. | M. | W. | M. | W. | M. | W. | M. | W. | M. | W. |
| 1850. | | | | | | | | | | | | |
| July . . . | 1 | 7 | 8 | 26 | 1 | 3 | 0 | 1 | 7 | 8 | 13 | 14 |
| August . . . | 1 | 9 | 9 | 24 | 1 | 1 | 0 | 2 | 6 | 6 | 12 | 12 |
| September . | 6 | 7 | 12 | 35 | 7 | 6 | 1 | 2 | 8 | 6 | 14 | 12 |
| October . . | 6 | 8 | 14 | 34 | 7 | 4 | 1 | 2 | 8 | 7 | 14 | 13 |
| November . . | 7 | 8 | 4 | 35 | 1 | 1 | 1 | 2 | 6 | 7 | 12 | 13 |
| December . . | 8 | 11 | 15 | 43 | 1 | 1 | 1 | 2 | 8 | 6 | 14 | 12 |

In the Report of 1851, it is stated that the total cured was greater than in 1850, viz. in 1850, 26, and in 1851, 35; or a per centage of 22·4 on the admissions of 1850, and 27·3 on those of 1851. During 1851 the amount of restraint is reported at 2·4 on every 100 patients in the house; and several reasons are given for being unable, in the then state of the house, to abolish restraint entirely. The number secluded amounted to 5·1 per cent. by day, and 13·1 per cent. by night. The following Table will show many other interesting facts. I regret that I have not any subsequent Report at hand. I am glad, however, to be able to state that since January of this year no restraint whatever has been employed in the asylum:—

### Table II.

*Showing the Admissions, Recoveries, and Deaths during* 1851, *with other particulars of interest.*

| | | | | M. | W. | Total. |
|---|---|---|---|---|---|---|
| In the Asylum, January 1st, 1851 . . . . | | | | 111 | 148 | 259 |
| Admitted during the year . . . . . . | | | | 67 | 61 | 128 |
| | | | | 178 | 209 | 387 |
| | M. | W. | Total. | | | |
| Discharged— | | | | | | |
| Cured . . . . . | 14 | 21 | 35 | | | |
| Improved . . . . | 5 | 2 | 7 | | | |
| Not improved . . . | 4 | 2 | 6 | | | |
| Died . . . . . . | 22 | 9 | 31 | | | |
| Total . . | 45 | 34 | 79 | 45 | 34 | 79 |
| Remaining in the Asylum at the end of the year . | | | | 133 | 175 | 308 |

| | M. | W. | Total. |
|---|---|---|---|
| Average number resident . . . . . . . | 127 | 170 | 297 |
| Sick, old, and unable to work . . . . . . | 39 | 68 | 107 |
| Engaged in work . . . . . . . . . | 87 | 102 | 190 |
| Attending Divine worship . . . . . . . | 55 | 59 | 114 |
| Dirty patients . . . . . . . . . | 8 | 12 | 20 |
| Violent ditto . . . . . . . . . | 15 | 34 | 49 |
| Under restraint . . . . . . . . . | 3 | 4 | 7 |
| Secluded by day . . . . . . . . . | 5 | 10 | 15 |
| Secluded by night . . . . . . . . | 14 | 25 | 39 |
| Attempts at suicide (unsuccessful) . . . . | 1 | 3 | 4 |
| Attempts at escapes (ditto) . . . . . . | 8 | 7 | 15 |
| Epileptics . . . . . . . . . . | 25 | 18 | 43 |
| Number of fits by day (9 a.m. to 9 p.m.) . . . | 1600 | 2415 | 4015 |
| Number of fits by night (9 p.m. to 9 a.m.) . . . | 1372 | 3435 | 4807 |

The asylum at Meerenberg has not been sufficiently long established to allow of much certainty in regard to statistics; and the first few years of an institution for the insane are always unfavourable to a large proportion of cures—a much larger number of incurable cases being in the first instance admitted. I have been favoured, however, by Dr. van Leeuwen with the following result. He says:—"In consequence of the moral system of treatment combined with a more regular medical treatment to which the recent cases are submitted—being about 50 out of 300 patients—and in which no mechanical coercion or restraint is practised as a general rule, about 50 per cent. of recent cases of insanity, and 25 per cent. of inveterate cases are restored to health and entire happiness."

The same writer has most kindly sent me a detailed account of an out-of-door entertainment or fête, which took place in the grounds of the asylum sometime ago. Such occurrences are so common in our English asylums that there is no longer much surprise experienced at the good order, &c. of the patients on these occasions. It is not, therefore, as anything novel or extraordinary that I give some report of the event here, but merely as the best proof that I can possibly adduce of the humane management of the establishment, and as a confirmation of the favourable impression I received on visiting it. It will also serve to illustrate Dutch manners and customs. To the good people in Holland the thing was perfectly surprising, and considered almost rash; a fact which must be borne in mind in estimating the great merit of the medical officers in proposing and carrying it out with success.

"The result of a regular vocal and instrumental concert" (writes Dr. van Leeuwen), "given for the first time at Meerenberg, quite surpassed our best expectations. Many days later did the patients continue to remember it gratefully, and several who previously always thought themselves imprisoned at Meerenberg gave up this terrible idea. A detailed account of it given in the papers excited a general interest and sympathy throughout the whole country, as the great bulk of the people in Holland had never yet heard of such a thing in a lunatic asylum, which they had always considered as identical with the mad-houses of the old system. Some benevolent persons assisted us with contributions, and one of the great statesmen and best national poets in Holland, Mr. M. C. van Hall, of Amsterdam, published a poem in memory of this philanthropic entertainment.

"As is usually every year the case in Holland on the 15th of April, the nightingales, the messengers of spring, appeared and delighted in hundreds the beautiful neighbourhood of Meerenberg and the village of Bloemendaal. As Easter Monday, a day on which the working-class in Holland spend the afternoon as much as possible in family parties, walks, and country feasts, was approaching, it seemed but right to prepare on that afternoon a similar recreation for the unhappy patients, who never since their deep affliction had enjoyed their former customs, and some of whom had a lively recollection of the old system of treatment, by which they had been confined to dungeons, and like brutes fastened by chains. To make the patients acquainted with the character and order of the feast, large '*Programmes of the Fête Champêtre, to be held in the afternoon of Easter Monday by the inhabitants of Meerenberg*,' were attached to the walls of the wards a few days before, and to every one who required it a ticket of admission was granted. The patients were filled with joy when they heard that large tents would be erected in the meadow to receive them, with a provision of Easter cakes, 1000 eggs, plenty of pickle, and bread and beer; that Punch and Judy would play; that there would be a shop kept by an old woman, boiling, selling, and distributing fresh hot oil-cakes; and that all kinds of games would be performed, and matches, for which prizes would be given to the winners; and, lastly, that a little band of music would attend the whole. The very anticipation of all these good things made them forget their sorrows!

"At four o'clock in the afternoon of Monday the great bell of the asylum called all the patients up to a large corridor, where they were arranged in the order directed by the programme. Their number amounted to 140, making with the attendants, friends, and visitors, about 250 persons. All being ready they went out, preceded by the band of music, through a broad beautiful avenue, behind the asylum to the field. Here they were received and addressed by their physician and friend, whose speech was listened to with great attention. After the address the male and female patients went to their respective tents, where they were treated with cake, eggs, and beer; then the matches and games began, varied as much as possible, and sometimes interrupted by the distribution of prizes, and by refreshments. I will not enter into a detailed description of the feast itself; it was similar to ordinary popular recreations, such as every one has witnessed once in his life; perhaps there was even more orderly conduct and less extravagance, owing to the behaviour of the attendants and the attention of the officers, who were masters of the ceremony. Certainly the refreshments and Punch and Judy caused the greatest delight.

"Only four out of the 140 patients required to be taken in on account of excitement and a desire to escape, and when at half-past six o'clock the bell of the asylum gave the signal that the feast was ending, all the patients followed the officers and attendants without any difficulty, and arranged themselves again in the order required to return home, where a supper of chocolate and cakes awaited them. After supper, the evening was spent in the same satisfactory manner by in-door entertainments, and the following night was as quiet as ever could have been wished."

The poem mentioned in the above account, as written by M. C. van Hall, ended with the words,—

"Come spring, come nightingales,
Come to Bloemendaal's sweet neighbourhood!"

In answer to this poetical effusion, Dr. van Leeuwen responded also in rhyme, and I have to thank a friend for having so successfully turned it into the following English verse:—

"Lured by your kindred notes, O greyhair'd bard of the city,
Joyous the nightingales throng to Bloemendaal's woods and gay parterres,
Filling its gardens around with many melodious echoes:
Mingling their song with your muse, loud thrills the rapturous concert,
Till Nature's chorus usurps the glory of Art's diapason;
Bringing a soothing charm to minds bowed down in distress;
Wooing awhile into peace the fiercest frenzies of madness!
That morn a hundred of those who have known that worst of afflictions,
Bent the knee unto Heaven with the prayer of a Christian household:
Now they wander afield to taste the fresh breath of the spring time
Thankful to Him who bestows such blessed season of respite.
Even to these whose life is so painfully darken'd with sorrow
Rises supreme over all that Faith whose power worketh wonders:
Joyful in freedom they roam, forgetting their bondage, while under
An eye that kindles with love, and brings to each heart consolation.
'Are these then those turbulent souls, whom rage and demoniac fury
'Urge with an impulse dire, as tho' sprung from the madness of Satan?'
Ah! no, let us soften with love the harshness of words breathing terror;
Restored to humanity's ranks, let us hail them once more as our fellows!
Behold their light-hearted joy! look around on their innocent gambols,
Threading the mazy dance—before the tents on the green sward;
Moved with joy is each heart by the genial impulse of spring time;

Lighted each face with a smile—as if to the season responsive!
Such the glad motions of spring—called forth by the nightingale's carol,
Kindling the spirit of love, and cheering the heart with its music.
Worthily have you sung, that by fostering care and attention
Glimmering sparks of the mind may be fann'd to a nobler existence.
Long then may Meerenberg thrive—long, long, may our Fatherland
witness,
How dear to the Hollander's heart are the tenderest ties of affection!"

Amsterdam.—Since the erection of the Meerenberg Asylum, the establishment formerly here and at Haarlem have been closed, and their insane removed to Meerenberg. The following is a statement showing the admissions, discharges, and deaths of the patients at these asylums from 1844 to 1849:—Admitted, 243 men; 288 women; total, 531. Cured, 189, or 35·5 per cent. of the admissions. Died, 150, being a mortality per cent. per annum on the mean number resident of 14·5.

There is a small asylum at Amsterdam for the Jews. It merits no particular description. I subjoin, however, a statement of the admissions, discharges, and deaths from 1844 to 1850 (inclusive). It exhibits a very low per centage of recoveries and a high rate of mortality. But it would not be fair to draw too decided an inference in so very small an asylum, from statistics extending over not more than seven years.

Table,

*Showing the Admissions, Recoveries, and Deaths at the Jews' Asylum, Amsterdam, from* 1844 *to* 1851:—

| | | | | M. | W. | Total. |
|---|---|---|---|---|---|---|
| Admitted during the seven years | | | | 46 | 52 | 98 |
| Remaining in the Asylum, January, 1844, | | | | 3 | 4 | 7 |
| | | | | 49 | 56 | 105 |
| | M. | W. | Total. | | | |
| Discharged recovered | 10 | 12 | 22 | | | |
| Discharged not recovered | 2 | 8 | 10 | | | |
| Died | 23 | 15 | 38 | | | |
| Of those in the Asylum, January, 1844— | | | | | | |
| Recovered | — | — | — | | | |
| Not recovered | 1 | — | 1 | | | |
| Died | 1 | 1 | 2 | | | |
| Total | 37 | 36 | 73 | | | |
| Total discharged and died in seven years | | | | 37 | 36 | 73 |
| Remaining in the Asylum, January, 1851 | | | | 12 | 20 | 32 |
| Average number resident during seven years | | | | 7·6 | 11·8 | 19·4 |
| | | | | M. | W. | Mean. |
| Proportion of the recoveries per cent. of the admissions | | | | 21·8 | 22·4 | 21·4 |
| Mean annual mortality per cent. resident | | | | 42·0 | 17·0 | 29·2 |

Utrecht.—"This asylum is situated in the town, and is, consequently, subjected to many disadvantages; the building is old also, and its plan has nothing particular to recommend it—a portion of it is about to be pulled down and rebuilt. Notwithstanding these disadvantages, however, there is at this asylum that which compensates (to a great extent) for architectural and local defects, a thorough desire to promote the comfort and cure of its inmates. I have seen Professor Schroeder van der Kolk, the visiting physician to the asylum; he is a man of great ability, and profoundly conversant with the subject of insanity; his ability is only equalled by his humanity, and to him, I believe, Holland owes more than to any other one man for the reformation which has been effected in the treatment of the insane. He introduced me to Dr. van Lith, the superintendent of the Utrecht Asylum, who kindly conducted me over it, and supplied me with the information I have obtained.

"The asylum provides for the insane, poor and rich, of the province of Utrecht —the population of which province is 149,347; the number of patients is 127, of whom 67 are men and 60 women. Hence it will be evident that a considerable number of insane must be unprovided for by this asylum, which is the only one for the province.

"About two-thirds of the patients are Protestants, and one-third Catholic. There are three classes as to payment, 25 being of the first or rich division (paying 66*l*. 12*s*. per annum), 27 being of the middle division, and paying half the above, and 75 being of the last or pauper class, and paying 15*l*. to 13*l*. 6*s*. There is no true classification of the patients in regard to their mental state. There are no separate rooms for the dirty and noisy; nor is there a chapel or schoolroom. The proportion of attendants is good, 17 to 127, or 1 to 7 and a fraction. One or two of them, however, are engaged in other work. Their wages appear to be, as at the other asylums I have visited, extremely low, the highest being 12*l*. a year.

"The pauper females were at dinner when I entered their room. I was informed that none were that day absent from table on account of noisy, violent conduct; they certainly looked extremely comfortable and clean, and were eating their dinner with great gusto.

"The day-rooms were comfortable—the airing-courts adjoining were small and filled with trees—they looked rather dark and gloomy, however. Owing to the situation of the asylum in the town, no room enjoys a free and extensive prospect. The bed-rooms were clean; the dormitories contained a large number; in one room I counted as many as 30 beds. The epileptics sleep in recesses in the walls, guarded by folding doors of open wood-work; this has an unpleasant effect, and is too much like a berth in a cabin.

"There was a room which gave satisfactory evidence of the occupation of the patients—a bazaar-room, in which I saw some fancy models of ships, houses, &c. very nicely executed. Indeed, in one way or another very few patients, not of the rich class, are unemployed. Some were busy tailoring, but few were at work during my visit, owing to its being a holiday.

"Neither Professor van der Kolk nor Dr. van der Lith subscribe unconditionally to the Non-Restraint System, but the amount of mechanical restraint practised is very small. On the day in which I visited the asylum I saw two patients restrained. Sometimes, a week passes without resorting to coercion, but on an average, Dr. van der Lith informed me, that probably one patient might be daily restrained. I was thoroughly satisfied, however, that a kind and humane spirit actuates the direction of this institution; and I can only hope that before very long an asylum may be provided *out* of the town of Utrecht, in which the same principles may be carried out towards the patients, but (owing to an improved site and building) with still greater success."

I may here introduce a short reference to the past history of this asylum extracted from the Appendix to Professor van der Kolk's "Oration," published 1837. To this I am able to add the statistics of the asylum from 1844 to 1851.

"Before 1831," says the Professor, "the insane of the lowest class alone were received into this asylum. These, shut up in narrow and stinking cells, were left in other respects to themselves, the sexes being entirely mixed. The numbers varied, but there were on an average between 20 and 30. From 1831 to the present time, the building has been more and more improved, so that even gardens have been added, a sufficient area for the free current of air afforded, adapted also for games and sundry occupations; and workshops have been built, in which various trades are performed by the patients. It was also provided that there should be open places in which those who were excited, noisy, and violent might be located; thus separated from others, they were able to enjoy the open air without danger to any one. * * * * *

"These and other improvements having been effected, the number of patients of every rank and place, annually sent to this asylum, was much increased; these were divided into three classes,—the rich, those of moderate means, and the poor. To each of these classes separate parts of the building were assigned, and gardens in which they were able to work, exercise themselves, or play, care being taken that there was a complete separation of the sexes. Those of the first class enjoy a single bedroom, ornamented in a manner similar to what they have been accustomed to at home. * * * * *

"In regard to the early condition of this asylum, it is to be remembered that the number of the insane which were annually admitted and cured was so small, that no comparison can be made between them, such as we have given in the following Table. For often one patient was admitted, and one was discharged cured, which led to an entirely false conclusion. From this cause, out of the number of patients who yearly entered our asylum, from the 1st day of January, for 30 years,—that is from 1800 to 1830,—we have taken the mean number, which is equal to 24; the number discharged cured during this period was 36, therefore 1·2 per annum. Consequently, the proportion is as 24 to 1·2. But for the last five years the mean number of patients was 55·4, and the number of the cured 17·6; consequently, the proportion is as 55·4 to 17·6. Hence it follows that the number of cures during the first 30 years of the century is to the number of cures during the last 5 years as 1·2 to 7·6, or as 3 to 19. * * * * * The contrast in the mortality during these is also equally striking. During the first 30 years of the century, the mortality calculated on the admissions was 35 per cent. During the last 5 years only 17 per cent."

The Professor adds the following Table of admissions and recoveries at the Utrecht asylum during five years:—

| Year. | Admissions. | | | Recoveries. | | | Per centage of Recoveries. |
|---|---|---|---|---|---|---|---|
| | M. | W. | Total. | M. | W. | Total. | |
| 1832-33 | 17 | 5 | 22 | 9 | 1 | 10 | 45 |
| 1833-34 | 32 | 12 | 44 | 14 | 0 | 14 | 31 |
| 1834-35 | 26 | 15 | 41 | 16 | 2 | 18 | 44 |
| 1835-36 | 32 | 21 | 53 | 15 | 5 | 20 | 38 |
| 1836-37 | 35 | 22 | 57 | 20 | 6 | 26 | 46 |
| 1832-37 | 142 | 75 | 217 | 74 | 14 | 88 | 40 |

It is singular that in the following Table, which embraces a subsequent period—namely, 1844 to 1851—the proportion of cures to the admissions is considerably less than it was in an earlier period of the institution:—

TABLE,

*Showing the Admissions, Recoveries, and Deaths at the Utrecht Asylum, from* 1844 *to* 1851.

| | M. | W. | T. | M. | W. | Total. |
|---|---|---|---|---|---|---|
| Admitted during the seven years . . . . . . . | | | | 173 | 119 | 292 |
| Remaining in the Asylum . . . . . . . . . | | | | 64 | 52 | 116 |
| | | | | 237 | 171 | 408 |
| Discharged— | | | | | | |
| Recovered . . . . . | 50 | 38 | 88 | | | |
| Not recovered . . . . | 35 | 20 | 55 | | | |
| Died . . . . . . . | 45 | 26 | 71 | | | |
| Of those in the Asylum, January, 1844— | | | | | | |
| Recovered . . . . . | 13 | 10 | 23 | | | |
| Not recovered . . . . | 8 | 9 | 17 | | | |
| Died . . . . . . . | 19 | 11 | 30 | | | |
| Total . . . | 170 | 114 | 284 | | | |
| Total discharged and died in seven years . . . . . . | | | | 170 | 114 | 284 |
| Remaining in the house, January, 1851 . . . . . . | | | | 67 | 57 | 124 |
| Average number resident during the seven years . . . | | | | 66 | 54·4 | 120·4 |

| | M. | W. | Mean. |
|---|---|---|---|
| Proportion of the recoveries per cent. of the admissions . . | 28·9 | 32 | 30·1 |
| Mean annual mortality per cent. resident . . . . . . | 13·7 | 9·5 | 11·6 |

ZUTPHEN.—"This place recals the name of the brave Sir Philip Sidney, for it was here that, after the close of the battle which cost him his life, Sir Philip Sidney, as generous as he was brave, refused the water offered him, and pointing to a common soldier near him, exclaimed, 'This man's necessity is greater than mine.' But I came here to visit a lunatic asylum, and not a battle field; and so will proceed at once to describe it.

"Accompanied by Dr. Ramaer, its physician, I went to the asylum, which is situated in the town of Zutphen itself, and is an old, irregularly built building, having nothing architecturally to recommend it. It provides for the insane of Gelderland, a province containing about 370,560 inhabitants. It contains 225 patients—about an equal number of men and women. The patients are arranged in three classes on the basis of payment—the scale is the same as at Utrecht. About one-third of the patients are Protestants, the remaining two-thirds Catholics and Jews.

"Great energy characterises Dr. Ramaer, and I believe he endeavours to provide for the comfort of the patients committed to his charge. Many of the patients were employed; I was pleased with the appearance of the tailor's room, in which many of the patients were actively at work, and engaged in lively conversation: there was a joiner's room also—and on the women's side of the house the patients were occupied with sewing, washing, &c. There is not, and hardly can be, out of door employment: it is grievous to think of the number of lunatic asylums which, simply by their locality, are unable to supply this important auxiliary to the endeavours of the superintendent to benefit the physical and the mental health of his patient. To some extent, no doubt, even in intra-mural asylums, this defect can be met by possessing land in the neighbourhood, and sending those who are suitable to work there. But to this plan there are many obvious objections, only less than having no land at all. The asylum has no school and no chapel; it was stated that the patients are taken to the chapels in the town.

"The baths are good; a douche is provided, but the bore is small. It is never used as a punishment by which to bully the patient into a confession of his delusions, but simply as a therapeutic agent. The ventilation is effected by windows, and is not so complete as is desirable. The day-rooms are heated by stoves, the bed-rooms are without; there is no apparatus for warming the house generally. To our English minds stoves never convey the same idea of comfort and cheerfulness as open fires. The airing courts are too crowded with trees in the Dutch asylums, and give one rather a gloomy impression in consequence; but if this is carried to excess in Holland, we in England perhaps go to the other extreme, and keep our airing courts too bare.

"With regard to restraint; at the time of my going round the house I saw only one man restrained; but there were two women; one, of the highest class, was confined by wrist straps; the other, a pauper, was securely fastened into a coercion chair, and wore besides a camisole. She was considerably excited, and was spitting in all directions. I should have very much preferred being in seclusion with my limbs free, had I been her, and as superintendent should have much preferred seeing her there. Dr. R. distinctly stated that restraint in her case had only been necessary for a few days, and would probably not be required much longer. The court for the noisy women contained about 30 patients,—many of them were boisterous, and would probably have been restrained in an inferior asylum; the dress was neat, and free from anything of a prison character, as some of our English strong dresses really are. Dr. R. has no padded room. There was a dormitory for the dirty patients, as well as those of cleanly habits, and owing to the number of beds, and the ventilation not being very good, I cannot doubt the rooms are often offensive. The number of patients in the other dormitories was far too large (as many as 30), and much crowded. One room which I entered, where there were several women patients ill in bed, was so offensive, that I could not have remained in it long without being sick.

"To these particulars I subjoin the following statistical information:—

TABLE,

*Showing the Admissions, Recoveries, and Deaths at the Zutphen Asylum, from* 1844 *to* 1851.

| | | | | M. | W. | Total. |
|---|---|---|---|---|---|---|
| Admitted during the seven years | | | | 228 | 211 | 439 |
| Remaining in the Asylum | | | | 44 | 29 | 73 |
| | M. | W. | Total. | 272 | 240 | 512 |
| Discharged recovered | 65 | 80 | 145 | | | |
| Discharged not recovered | 17 | 10 | 27 | | | |
| Died | 70 | 50 | 120 | | | |
| Of those in the Asylum, January, 1844— | | | | | | |
| Recovered | 3 | 3 | 6 | | | |
| Not recovered | 2 | 4 | 6 | | | |
| Died | 15 | 9 | 24 | | | |
| Total | 172 | 156 | 328 | | | |
| Total discharged and died in seven years | | | | 172 | 156 | 328 |
| Remaining in the house, January, 1851 | | | | 100 | 84 | 184 |
| Mean number resident during seven years | | | | 81·4 | 72· | 153·4 |

| | M. | W. | Mean. |
|---|---|---|---|
| Proportion of the recoveries per cent. of the admissions | 37·9 | 28·5 | 33·03 |
| Mean annual mortality per cent. resident | 14·8 | 6·9 | 10·8 |

"Dr. R. constantly finds a much larger proportion of noisy patients among the women than the men—at present there are nearly twice as many of the former more or less excited. On mentioning this to ———, as a reproach on the gentler sex, she argued that it was natural to suppose women would be more noisy than men when insane, because they are so much less so when they are sane!

"At Zutphen I met with the superintendent of the Deventer Asylum, formerly under the charge of Dr. Everts. I had intended to have visited it, but several circumstances, and the necessity for hastening on to Siegburg, induced me to omit Deventer, as also the colonies of criminals at Ryssel, which I had much desired to visit. I subjoin, however, the following statistical information in regard to the Deventer Asylum from 1844 to 1851. It exhibits the highest per centage of cures of any asylum in Holland:—

TABLE,

*Showing the Admissions, Recoveries, and Deaths at the Deventer Asylum, from* 1844 *to* 1851.

| | | | | M. | W. | Total. |
|---|---|---|---|---|---|---|
| Admitted during the seven years | | | | 146 | 103 | 249 |
| Remaining in the Asylum, January, 1844 | | | | 69 | 47 | 116 |
| | | | | 215 | 150 | 365 |
| | M. | W. | Total. | | | |
| Discharged recovered | 55 | 46 | 101 | | | |
| Discharged not recovered | 17 | 13 | 30 | | | |
| Died | 29 | 16 | 45 | | | |
| Of those in the Asylum, January, 1844— | | | | | | |
| Recovered | 8 | 7 | 15 | | | |
| Not recovered | 6 | 8 | 14 | | | |
| Died | 24 | 14 | 38 | | | |
| Total | 139 | 104 | 243 | | | |
| Total discharged and died in seven years | | | | 139 | 104 | 243 |
| Remaining in the house, January, 1851 | | | | 76 | 46 | 122 |
| Average number resident during the seven years | | | | 79·4 | 46·4 | 126 |

| | M. | W. | Mean. |
|---|---|---|---|
| Proportion of the recoveries per cent. of the admissions | 37·7 | 44·6 | 40·6 |
| Mean annual mortality per cent. resident | 9·4 | 9·2 | 9·3 |

"I have now seen the principal Dutch asylums, and shall leave Holland to-morrow. In reviewing their condition generally, it will be evident that they are not yet perfect; a great work of reform, however, has been commenced, and considering the period of time which has elapsed, I think that the progress made in the amelioration of the condition of the insane is highly satisfactory. One noble institution has been reared, of which any country might justly be proud, an institution which, itself the indication and result of the reform in Holland, will in its turn act as the nucleus of an extended improvement in the management of the Dutch asylums. It will prove—it has proved already—the truth of those principles in the treatment of the insane which are now so universally admitted in our own country, but which to many in Holland, a few years back, were comparatively little known. And if I am not greatly mistaken, late as Holland has been in reforming the state of her asylums, she will ere long advance to a point beyond that arrived at by some Continental countries which began the work of reformation before her. She has ever been in advance of many other countries in her philanthropic institutions, and it was only because she failed to appreciate the character of the lunatic that she neglected him; his true character recognised, she will with a firm purpose and unrelaxed energy pursue the work which she has so nobly begun."

The chief defects which still remain may be briefly summed up as follows:—1st. The non-residence in many asylums of any medical officer or well-educated non-medical superintendent. 2nd. The situation of most of the asylums—in the heart of the town—and the consequent want of out-of-door employment. 3rd. The substitutes for mechanical restraint are not sufficiently introduced; and the provision for dirty and violent patients incomplete. 4th. The classification of the patients in regard to mental condition is far from perfect. 5th. Either the number of the Commissioners should be increased, or they should be able to devote their time exclusively to the subject.

The present Commissioners, however, have worked laboriously at their office, and have obtained much valuable statistical information, from which I have prepared the following Tables. They afford important results relative to the admissions, discharges, &c., of the patients. I may here briefly remark upon the most salient of these. In taking any single institution, and comparing its statistics with another, we are exposed to many fallacies arising from the possibility of different circumstances in each. But this source of error is less likely to occur when we take the whole or a large number of the asylums of a country, and examine the grand results, not only as facts of independent interest, but as compared with the results of a large number of asylums in other countries. We are able also, in the present Tables, to distinguish the cures of the patients admitted after a given period from the cures of those previously in the establishment. Hence the per centage of cures on the admissions is exact. An interesting fact in the present case is apparent, from being able to make this distinction. Of the 837 in the asylums in 1844, only 11·9 per cent. were cured up to 1851; while of the admissions which took place between 1844-51, 32·4 per cent. were cured. We are aware that we are here comparing two different things—the per centage on the admissions and the per centage on the number in the house in a certain year; but the allowance to be made for this is comparatively small, and still leaves a large actual difference. This is principally to be explained, I imagine, by the large number of incurable cases which must have been in the asylums at that time (1844); and shows that they have become, as it was intended they should, *medical* asylums, instead of mere places of confinement.

With regard to the recoveries of patients since 1844, it will be seen from Table I., that of the total number of patients (3087) admitted into the Dutch asylums, 1000 were discharged "recovered;" giving a per centage of 32·4. It is interesting to compare this result, as Table II.* will enable the reader to do, with the recoveries, calculated on the same principle, of the insane in various asylums in England and other countries. It may be well to premise, that the comparison can only justly be made between the asylums in Holland and those asylums which receive epileptic and incurable cases; and also, that the Holland asylums receive all classes as regards pecuniary con-

* The figures in this Table, introduced in order to make a comparison, are taken from Dr. Thurnam's "Statistics of Insanity."

ditions. Thus, in 1850, 153 were of the superior, 36 of the middle, and 1074 of the lowest class: hence, about five-sixths were of the pauper class. Now, comparing the above per centage of recoveries with an average of 6 asylums in England receiving both private and pauper patients (about one-third of the former), we observe that a considerably larger proportion recovered in the 6 English asylums. On the other hand, in the metropolitan licensed houses, during 4 years (containing more than one-half paupers), the proportion of recoveries was as low as 25·65 per cent., being 6·75 below the per centage in Holland. Again, what is remarkable, at Siegburg, where only curable cases are admitted, only 30·73 per cent. recovered. Considering the condition from which the asylums in Holland have so recently emerged, I think the proportion of recoveries as high as could be expected; it is probable that for the succeeding decennial period, it will be raised nearly 10 per cent. Dr. Thurnam, in his "Statistics," states that he considers, under ordinary circumstances, a proportion much below 40 per cent. to be low, and one much exceeding 45 per cent. to be high.

In regard to the mortality, this I have calculated on the mean number annually resident in the Dutch asylums. It is certainly high. The average of six English asylums receiving private and pauper patients, exhibits a mean annual mortality of 10·46; the mortality, however, of nine asylums in England, receiving paupers only, was 13·88; and that of the metropolitan licensed houses (1839-43), 14·68.

### Table I.

*Showing the Admissions, Discharges, and Deaths of Patients in the Dutch Asylums, during seven years* (1844-51).

| | | | | M. | F. | Total. |
|---|---|---|---|---|---|---|
| Remaining in the Asylums, January 1st, 1844 | | | | 424 | 413 | 837 |
| Admitted during seven years (1844-51) | | | | 1590 | 1497 | 3087 |
| | | | | 2014 | 1910 | 3924 |
| | M. | F. | Total. | | | |
| Discharged— | | | | | | |
| (a) Of those in the Asylums, January, 1844. | | | | | | |
| Recovered | 56 | 44 | 100 | | | |
| Not recovered | 38 | 42 | 80 | | | |
| Died | 159 | 165 | 324 | | | |
| (b) Of those subsequently admitted. | | | | | | |
| Recovered | 477 | 523 | 1000 | | | |
| Not recovered | 191 | 152 | 343 | | | |
| Died | 474 | 327 | 801 | | | |
| Total | 1395 | 1253 | 2648 | | | |
| Total discharged and died during seven years | | | | 1395 | 1253 | 2648 |
| Remaining January 1st, 1851 | | | | 619 | 657 | 1276 |
| Average number resident during the seven years | | | | 608 | 502 | 1110 |

| | M. | F. | Mean. |
|---|---|---|---|
| Proportion of the recoveries per cent. of the admissions | 30· | 34·9 | 32·4 |
| Mean annual mortality per cent. resident | 15·1 | 13·9 | 14·5 |

### Table II.

*Exhibiting the Comparative Statistics of various Asylums in Great Britain, Holland, France, Germany, and America.*

| Name and Description of Asylums. | Remaining under Care, Jan. 1st, 1844. | Numbers Admitted. | Numbers Recovered. | Numbers Died. | Proportion of Recoveries per cent. of Admissions. | Mean Annual Mortality per cent. Resident. |
|---|---|---|---|---|---|---|
| Average of eleven Dutch Asylums, for pauper and private patients . . | 837 | 3087 | 1000 | 1125 | 32·40 | 14·5 |
| Average of nine English County Asylums, receiving paupers only . . | 3273 | 15548 | 5746 | 4551 | 36·95 | 13·88 |
| Average of six English County Asylums, receiving private and pauper patients . . . . . | 1127 | 7738 | 3627 | 1256 | 46·87 | 10·46 |
| Average of Metropolitan Licensed Houses, 1839-43 (more than half paupers) | 1827 | 5850 | 1501 | 1209 | 25·65 | 14·68 |
| The York Asylum (one-third paupers), 1814-44 . . | 157 | 1375 | 475 | 297 | 34·54 | 7·24 |
| The York Retreat, 1796-1847 . . . . . . . . | 84 | 593 | 292 | 141 | 49·24 | 4·74 |
| Average of seven Scotch chartered Asylums (more than half paupers) . . | 1324 | 7130 | 3021 | 931 | 42·37 | 7·52 |
| Average of ten (pauper) Irish Asylums . . . | 2147 | 10255 | 4957 | 1891 | 48·33 | 8·7 |
| Average of five American Asylums (private and pauper patients) . . | 640 | 8675 | 4062 | 688 | 46·82 | 9·56 |
| Charenton, 1826-33 (private patients) . . . . . | ... | 1557 | 518 | 546 | 33·26 | 14·96 |
| Siegburg, 1825-40 (only curable patients) . . | ... | 1129 | 347 | 161 | 30·73 | 7·4 |

### Table III.

*Showing the Ages of Patients in each Decennial Period admitted into the Dutch Asylums in* 1850*; also the Form of the Disorder.*

| Decennial Period. | MEN. | | | | | | | WOMEN. | | | | | | | Total of Men and Women. |
|---|---|---|---|---|---|---|---|---|---|---|---|---|---|---|---|
| | Mania. | Monomania. | Melancholia. | Dementia. | Idiotcy. | Epilepsy. | Total. | Mania. | Monomania. | Melancholia. | Dementia. | Idiotcy. | Epilepsy. | Total. | |
| Under 10 . . . . | ... | ... | ... | ... | ... | 2 | 2 | ... | ... | ... | 1 | ... | ... | 1 | 3 |
| From 10 to 20 . . | 4 | 2 | ... | 2 | 2 | 4 | 14 | 4 | 1 | ... | ... | 1 | ... | 6 | 20 |
| „ 20 to 30 . . | 19 | 9 | 5 | 11 | 1 | 10 | 55 | 20 | 3 | 12 | 6 | 4 | 5 | 50 | 105 |
| „ 30 to 40 . . | 19 | 12 | 8 | 12 | 4 | 2 | 57 | 23 | 5 | 10 | 8 | ... | 1 | 47 | 104 |
| „ 40 to 50 . . | 20 | 14 | 12 | 11 | ... | 1 | 58 | 18 | 10 | 13 | 12 | 2 | ... | 55 | 113 |
| „ 50 to 60 . . | 8 | 4 | 6 | 5 | ... | ... | 23 | 19 | 4 | 12 | 9 | ... | ... | 44 | 67 |
| „ 60 to 70 . . | 2 | 2 | 5 | 2 | ... | ... | 11 | 7 | 5 | 4 | 4 | ... | ... | 20 | 31 |
| Above 70 . . . . | 1 | ... | ... | ... | ... | ... | 1 | 1 | ... | 1 | 12 | ... | ... | 14 | 15 |
| Total . . . | 73 | 43 | 36 | 43 | 7 | 19 | 221 | 92 | 28 | 52 | 52 | 7 | 6 | 237 | 458 |

### Table IV.

*Showing of the* 378 *discharged in* 1850, *the Ages in Decennial Periods, and the Form of the Disorder.*

| Ages of the Patients Discharged and Died. | Men. | | | | | | Women. | | | | | | Total Discharged and Died. |
|---|---|---|---|---|---|---|---|---|---|---|---|---|---|
| | Dead. | Not Insane. | Not Improved. | Improved. | Cured. | Total. | Dead. | Not Insane. | Not Improved. | Improved. | Cured. | Total. | |
| Under 10 . . . . . . . | ... | ... | ... | ... | 1 | 1 | ... | ... | ... | ... | ... | 1 | 2 |
| 10 to 20 . . . . . . . | 6 | 1 | 1 | ... | 1 | 9 | 3 | 1 | 1 | ... | 2 | 7 | 16 |
| 20 to 30 . . . . . . . | 8 | 1 | 6 | 6 | 20 | 41 | 10 | ... | 5 | 1 | 22 | 38 | 79 |
| 30 to 40 . . . . . . . | 19 | 2 | 3 | 3 | 24 | 51 | 17 | ... | ... | 2 | 24 | 43 | 94 |
| 40 to 50 . . . . . . . | 15 | 1 | 2 | 8 | 19 | 45 | 6 | ... | 4 | 4 | 18 | 32 | 77 |
| 50 to 60 . . . . . . . | 17 | ... | 2 | ... | 15 | 34 | 11 | 2 | 1 | ... | 16 | 30 | 64 |
| 60 to 70 . . . . . . . | 12 | ... | ... | 1 | 6 | 19 | 10 | 1 | 1 | ... | 5 | 17 | 36 |
| Above 70 . . . . . . . | 2 | ... | ... | ... | ... | 2 | 7 | ... | ... | 1 | ... | 8 | 10 |
| Total . . . | 79 | 5 | 14 | 18 | 86 | 202 | 64 | 4 | 12 | 8 | 88 | 176 | 378 |

### Table V.

*Showing the Period of Residence in the Asylums of those who recovered and died in* 1850.

